# Sex-associated differences in lesion delivery and atrial fibrillation recurrence after cryoballoon ablation

**DOI:** 10.1093/europace/euag189

**Published:** 2026-09-04

**Authors:** Emanuel Heil, Matthias Bock, Julia Knocks, Jan Paul Rudolph, Jin-Hong Gerds-Li, Frank Heinzel, Philipp Attanasio, Abdul Shokor Parwani, Hendrik Boldt, Florian Blaschke, Doreen Schöppenthau, Julia Christina Lueg, Till Althoff, Nikolaos Dagres, Verena Tscholl, Gerhard Hindricks, Felix Hohendanner

**Affiliations:** Deutsches Herzzentrum der Charité, Department of Cardiology, Angiology, and Intensive Care Medicine, Charitéplatz 1, Berlin 10117, Germany; Charité—Universitätsmedizin Berlin, corporate Member of Freie Universität Berlin and Humboldt-Universität zu Berlin, Charitéplatz 1, Berlin 10117, Germany; DZHK (German Centre for Cardiovascular Research), Partner Site Berlin, Berlin, Germany; Deutsches Herzzentrum der Charité, Department of Cardiology, Angiology, and Intensive Care Medicine, Charitéplatz 1, Berlin 10117, Germany; Charité—Universitätsmedizin Berlin, corporate Member of Freie Universität Berlin and Humboldt-Universität zu Berlin, Charitéplatz 1, Berlin 10117, Germany; Sana Klinikum Lichtenberg, Klinik Innere Medizin II: Kardiologie, Fanningerstraße 32, Berlin 10365, Germany; Sana Klinikum Lichtenberg, Klinik Innere Medizin II: Kardiologie, Fanningerstraße 32, Berlin 10365, Germany; Deutsches Herzzentrum der Charité, Department of Cardiology, Angiology, and Intensive Care Medicine, Charitéplatz 1, Berlin 10117, Germany; Charité—Universitätsmedizin Berlin, corporate Member of Freie Universität Berlin and Humboldt-Universität zu Berlin, Charitéplatz 1, Berlin 10117, Germany; DZHK (German Centre for Cardiovascular Research), Partner Site Berlin, Berlin, Germany; Städtisches Klinikum Dresden, 2. Medizinische Klinik, Friedrichstraße 41, Dresden 01067, Germany; Deutsches Herzzentrum der Charité, Department of Cardiology, Angiology, and Intensive Care Medicine, Charitéplatz 1, Berlin 10117, Germany; Charité—Universitätsmedizin Berlin, corporate Member of Freie Universität Berlin and Humboldt-Universität zu Berlin, Charitéplatz 1, Berlin 10117, Germany; DZHK (German Centre for Cardiovascular Research), Partner Site Berlin, Berlin, Germany; Deutsches Herzzentrum der Charité, Department of Cardiology, Angiology, and Intensive Care Medicine, Charitéplatz 1, Berlin 10117, Germany; Charité—Universitätsmedizin Berlin, corporate Member of Freie Universität Berlin and Humboldt-Universität zu Berlin, Charitéplatz 1, Berlin 10117, Germany; DZHK (German Centre for Cardiovascular Research), Partner Site Berlin, Berlin, Germany; Deutsches Herzzentrum der Charité, Department of Cardiology, Angiology, and Intensive Care Medicine, Charitéplatz 1, Berlin 10117, Germany; Charité—Universitätsmedizin Berlin, corporate Member of Freie Universität Berlin and Humboldt-Universität zu Berlin, Charitéplatz 1, Berlin 10117, Germany; DZHK (German Centre for Cardiovascular Research), Partner Site Berlin, Berlin, Germany; Deutsches Herzzentrum der Charité, Department of Cardiology, Angiology, and Intensive Care Medicine, Charitéplatz 1, Berlin 10117, Germany; Charité—Universitätsmedizin Berlin, corporate Member of Freie Universität Berlin and Humboldt-Universität zu Berlin, Charitéplatz 1, Berlin 10117, Germany; DZHK (German Centre for Cardiovascular Research), Partner Site Berlin, Berlin, Germany; Deutsches Herzzentrum der Charité, Department of Cardiology, Angiology, and Intensive Care Medicine, Charitéplatz 1, Berlin 10117, Germany; Charité—Universitätsmedizin Berlin, corporate Member of Freie Universität Berlin and Humboldt-Universität zu Berlin, Charitéplatz 1, Berlin 10117, Germany; DZHK (German Centre for Cardiovascular Research), Partner Site Berlin, Berlin, Germany; Deutsches Herzzentrum der Charité, Department of Cardiology, Angiology, and Intensive Care Medicine, Charitéplatz 1, Berlin 10117, Germany; Charité—Universitätsmedizin Berlin, corporate Member of Freie Universität Berlin and Humboldt-Universität zu Berlin, Charitéplatz 1, Berlin 10117, Germany; DZHK (German Centre for Cardiovascular Research), Partner Site Berlin, Berlin, Germany; Deutsches Herzzentrum der Charité, Department of Cardiology, Angiology, and Intensive Care Medicine, Charitéplatz 1, Berlin 10117, Germany; Charité—Universitätsmedizin Berlin, corporate Member of Freie Universität Berlin and Humboldt-Universität zu Berlin, Charitéplatz 1, Berlin 10117, Germany; DZHK (German Centre for Cardiovascular Research), Partner Site Berlin, Berlin, Germany; Deutsches Herzzentrum der Charité, Department of Cardiology, Angiology, and Intensive Care Medicine, Charitéplatz 1, Berlin 10117, Germany; Charité—Universitätsmedizin Berlin, corporate Member of Freie Universität Berlin and Humboldt-Universität zu Berlin, Charitéplatz 1, Berlin 10117, Germany; DZHK (German Centre for Cardiovascular Research), Partner Site Berlin, Berlin, Germany; Deutsches Herzzentrum der Charité, Department of Cardiology, Angiology, and Intensive Care Medicine, Charitéplatz 1, Berlin 10117, Germany; Charité—Universitätsmedizin Berlin, corporate Member of Freie Universität Berlin and Humboldt-Universität zu Berlin, Charitéplatz 1, Berlin 10117, Germany; DZHK (German Centre for Cardiovascular Research), Partner Site Berlin, Berlin, Germany; Deutsches Herzzentrum der Charité, Department of Cardiology, Angiology, and Intensive Care Medicine, Charitéplatz 1, Berlin 10117, Germany; Charité—Universitätsmedizin Berlin, corporate Member of Freie Universität Berlin and Humboldt-Universität zu Berlin, Charitéplatz 1, Berlin 10117, Germany; DZHK (German Centre for Cardiovascular Research), Partner Site Berlin, Berlin, Germany; Deutsches Herzzentrum der Charité, Department of Cardiology, Angiology, and Intensive Care Medicine, Charitéplatz 1, Berlin 10117, Germany; Charité—Universitätsmedizin Berlin, corporate Member of Freie Universität Berlin and Humboldt-Universität zu Berlin, Charitéplatz 1, Berlin 10117, Germany; DZHK (German Centre for Cardiovascular Research), Partner Site Berlin, Berlin, Germany

**Keywords:** Atrial fibrillation, Pulmonary vein isolation, Cryoballoon ablation, Cryothermal exposure, Sex differences, Precision medicine

## Introduction

Atrial fibrillation (AF) is more prevalent in men, but women have greater symptom burden, more advanced remodelling, and more non-pulmonary-vein triggers. After catheter ablation, women show higher rates of durable pulmonary vein isolation (PVI), recurrence, and adverse events.^1–3^ While anatomical differences may contribute, it remains unclear whether ablation-dosing is comparable between sexes.^4,5^

## Methods

Under ethics approval (EA2/102/24), 2137 consecutive patients undergoing fluoroscopy-guided first-time PVI with a fixed-size 28-mm cryoballoon at six German hospitals were included. Phrenic nerve monitoring was compound motor action potential (CMAP)-guided during right-sided applications. Oesophageal temperature monitoring was operator-dependent and non-standardized. Except for CMAP-related safety termination, freezes were ended time-based. Recurrence was defined as arrhythmia ≥30 s or physician-documented recurrence. Patients without 12-month follow-up were excluded. Clinical and imaging data were extracted, with left atrial size standardized to diameter (LAD) using validated conversions.^6^ Temperature-time curves were analysed to derive cumulative cryothermal exposure. Effective cryothermal dose (CTD) was defined as the area under the temperature-time curve below −40°C, a pragmatic threshold based on ex vivo myocardial injury data and expected probe-to-surface temperature differences.^7^ Natural logarithmic transformation (ln[CTD]) accounted for diminishing effects at lower temperatures. Curves not meeting predefined criteria (typical profile, duration >90 s, minimum temperature ≤−10°C) were excluded. Continuous and categorical variables were analysed using standard tests.

Recurrence predictors were assessed using cumulative CTD-focused multivariable logistic regressions adjusted for clinical covariates and centre effects, evaluated by calibration and bootstrap resampling. To assess whether sex-associated procedural information was present in non-aggregated temperature-time curves, we trained multiple-instance learning (MiLM) models. Patients were represented as padded and masked bags of resampled application curves. Models were trained on five centres (*n* = 11 707 curves) and validated in one held-out centre (*n* = 724 curves). Patient-level cross-validation, fold-wise calibration, and ensemble averaging were used for internal evaluation. A conventional logistic-regression comparator using prespecified cryothermal summary variables benchmarked the full-curve signal. Feature importance was assessed by permutation-based change in Brier score.

## Results

**Figure 1:**
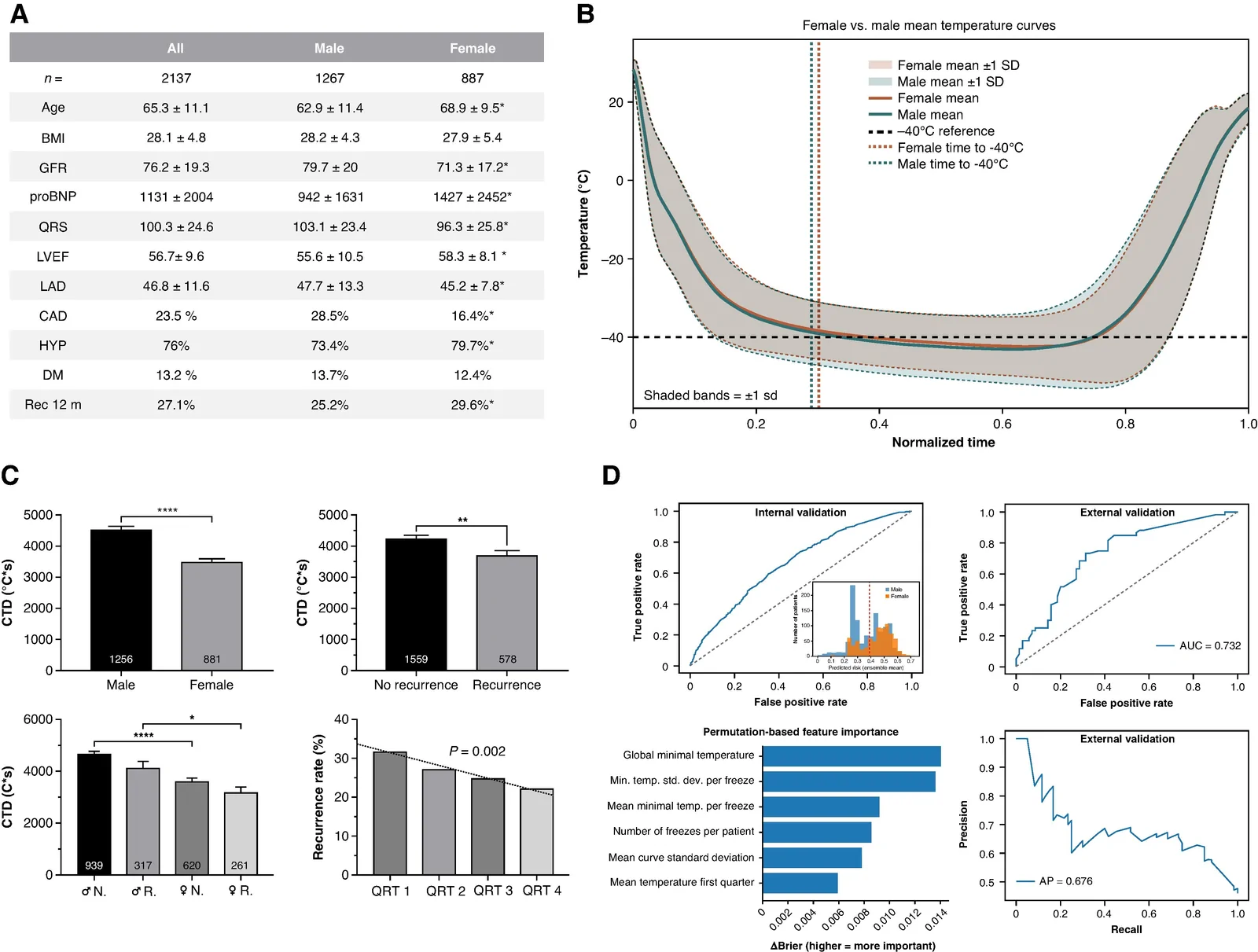
(*A*) Baseline characteristics of the overall cohort and stratified by sex. Age in years; BMI, body mass index in kg/m^2^; CAD, coronary artery disease; DM, diabetes mellitus; GFR, glomerular filtration rate in mL/min/1.73 m^2^; HYP, arterial hypertension; LAD, estimated left atrial diameter in mm; LVEF, left ventricular ejection fraction in %; proBNP, pro-B-type natriuretic peptide in ng/L; QRS, QRS duration in ms; and Rec12 m, recurrence within 12 months. Asterisks indicate significant differences between sexes. (*B*) Sex-specific mean cryothermal temperature curves with standard deviation over normalized application time; individual outliers were reviewed. (*C*) Cryothermal dose by sex and recurrence status, and recurrence rates across dose quartiles. CTD, cryothermal dose in °C·s; QRT, quartile. (*D*) Multiple-instance learning model performance for sex classification, including internal and external validation and permutation-based feature importance. Temp, temperature; min., minimum.

Patients showed largely comparable baseline characteristics (*Figure 1A*) and had persistent AF in 55% (♂ 56% vs. ♀ 52%; *P* = 0.058). Within 12 months, arrhythmia recurrence occurred in 27% of patients with a higher frequency in women (♂ 25.2% vs. ♀ 29.6%, *P* = 0.025).

On average, 6 ± 2 temperature recordings passed criteria for clinically meaningful cryothermal exposure. Men demonstrated modestly longer therapy (♂ 1582 ± 712 vs. ♀ 1478 ± 652 s, *P* < 0.001) and freeze-duration (*P* = 0.030) as well as lower minimal (♂ −55 ± 6 vs. ♀ −53 ± 6°C, *P* < 0.001) and mean temperatures during freeze (♂ −43 ± 5 vs. ♀ −42 ± 5°C, *P* < 0.001). Consequently, men exhibited greater freeze exposure, with longer time below −40°C (♂ 612 ± 390 vs. ♀ 543 ± 334 s, *P* < 0.001) and higher CTD (♂ 4512 ± 3956 vs. ♀ 3487 ± 3093, *P* < 0.001) and ln(CTD) (♂ 6.21 ± 0.77 vs. ♀ 6.13 ± 0.70, *P* = 0.009). Maximal (*P* = 0.042) and mean temperature drop rate (♂ −1.56 ± 0.28 vs. ♀ −1.64 ± 0.30°C/s, *P* < 0.001) were steeper in women (*Figure 1B, 1C*).

The multivariable logistic-regression model (*n* = 1509; 26.7% 12-month recurrence), including sex, age, LVEF, LAD, ln(CTD), treatment centre, and comorbidities, was statistically significant (*P* < 0.001) and demonstrated good calibration. Bootstrap validation confirmed robustness [95% bias-corrected and accelerated (BCa) confidence interval (CI) −0.495 to −0.182, *P* < 0.001].

Higher ln(CTD) (OR 0.717, 95% CI 0.612–0.838, *P* < 0.001) and higher LVEF (OR 0.979 per %, 95% CI 0.968–0.991, *P* < 0.001) were independently associated with a lower recurrence risk. A graded dose–response relationship across CTD quartiles was observed (*P* = 0.002). Larger LAD (OR 1.013 per mm, 95% CI 0.999–1.028, *P* = 0.067) and greater age showed a trend towards higher recurrence risk (OR 1.011 per year, 95% CI 0.999–1.023, *P* = 0.062). Other covariates, including sex (OR 1.012, 95% CI 0.784–1.307, *P* = 0.928), were not independently contributing to recurrence. No sex-by-thermal-dose interaction was observed (*P* = 0.699). Sensitivity analyses using alternative thresholds showed consistent results for CTD below −30°C (OR 0.729, *P* = 0.003), whereas a more restrictive threshold of −50°C, which is below the mean freeze temperature of −42.2°C, was not significant (OR 0.948, *P* = 0.157).

In exploratory full-curve analysis, MiLM identified sex-associated information in cryothermal curves with modest internal discrimination [ensemble area-under-the-curve (AUC) 0.66; Brier 0.21] and similar external performance (AUC 0.73, 95% CI 0.64–0.82; Brier 0.22). At the optimized threshold, performance was balanced internally (accuracy 0.68, sensitivity 0.73, specificity 0.69, F1 0.69) and externally (accuracy 0.64, sensitivity 0.66, specificity 0.61, F1 0.64). A conventional logistic-regression comparator based on prespecified cryothermal summary variables achieved lower discrimination (AUC 0.607, 95% CI 0.581–0.633), suggesting that conventional temperature-dose metrics captured part, but not all, of this signal. Importance analysis indicated that minimum-temperature metrics contributed most strongly to model performance (*Figure 1D*). These findings support the presence of sex-associated procedural patterns.

## Discussion

Higher CTD showed a consistent, graded association with lower recurrence, supporting a dose–response relationship.^8^ Lower CTD was associated with higher recurrence and attenuated the unadjusted association between female sex and recurrence, suggesting that procedural exposure may have partly contributed to observed sex-related differences in outcome. Exploratory full-curve MiLM supported reproducible sex-associated procedural patterns.

Women received lower effective cryothermal exposure despite comparable procedural endpoints. However, although CTD may be modifiable, increasing CTD may pose risks. The observed sex-related difference may reflect variation in balloon–tissue coupling due to anatomical or procedural factors.^9,10^ Men have been reported to exhibit greater anterior and septal atrial wall thickness, larger pulmonary vein diameters and cross-sectional areas, and longer pulmonary vein sleeves. Pulmonary vein wall thickness has not been directly studied in this context. Comparable temperature-decline dynamics argue against a dominant isolated role of intrinsic tissue characteristics, but do not exclude subtler anatomical or procedural mechanisms.

## Limitations

Retrospective design, centre variability, and incomplete procedural-strategy capture introduce potential bias and residual confounding. CTD should be interpreted as a cryothermal exposure marker, not as a validated tissue-level lesion metric or directly titratable treatment target. Vein-specific applications, operator strategy, occlusion quality, and direct tissue characteristics were unavailable, limiting mechanistic interpretation. Follow-up reflected routine, Holter-ECG-based care. Therefore, asymptomatic recurrence may have been underestimated, and AF burden could not be assessed.

## Conclusion

Women exhibited higher recurrence rates despite similar procedural endpoints. This difference was not independently attributable to sex after adjustment and was associated with lower cryothermal exposure. Exploratory full-curve modelling supported sex-associated procedural patterns. Strategies assessing effective lesion formation may improve outcomes.

## Data Availability

The data underlying this article will be shared at a reasonable request by the corresponding author.
